# Objective measurement of sedentary behavior: impact of non-wear time rules on changes in sedentary time

**DOI:** 10.1186/s12889-015-1847-6

**Published:** 2015-05-23

**Authors:** Xanne Janssen, Laura Basterfield, Kathryn N. Parkinson, Mark S. Pearce, Jessica K. Reilly, Ashley J. Adamson, John J. Reilly, the Gateshead Millennium Study Core Team

**Affiliations:** School of Psychological Sciences and Health, University of Strathclyde, Glasgow, Scotland G1 1QN UK; Institute of Health & Society, Human Nutrition Research Centre, Newcastle University, Newcastle Upon Tyne, UK; Institute of Health & Society Newcastle, Sir James Spence Institute of Child Health, Royal Victoria Infirmary, Newcastle University, Newcastle Upon Tyne, UK

**Keywords:** Accelerometry, Child, Adolescent, Sedentary behavior, Non-wear time

## Abstract

**Background:**

Accelerometry non-wear time rules might affect sedentary time, and the associations with health outcomes such as adiposity. However, the exact effect of different non-wear time rules on sedentary time and reported changes in sedentary time is unknown. This study evaluated the effect of different accelerometry non-wear time rules on sedentary time and changes in sedentary time from age 9–12 years.

**Methods:**

Accelerometry data were collected as part of the Gateshead Millennium Birth Cohort study. Participants were 9.3 (±0.4) years at baseline (*n* = 517) and 12.5 (±0.3) years at follow-up (*n* = 440). Sedentary time was defined using an accelerometry cut-point of 25 counts per 15 s. Non-wear time was defined using manual data reduction (the reference method) and 10 min, 20 min and 60 min consecutive zeros. Differences between methods were analyzed using repeated measures ANOVA with Bonferroni post-hoc analyses.

**Results:**

Mean daily sedentary time at age 9 ranged from 364 min per day to 426 min using the 10 min and 60 min rule, respectively (*p* < 0.05). At 12 years, mean daily sedentary times ranged from 424 min to 518 min (*p* < 0.05). Mean changes in daily sedentary time over the three years ranged from 60 min to 93 min using the 10 min and 60 min rule, respectively (*p* < 0.05). When adjusting for wear time, differences in average sedentary time between methods decreased from 62 min to 27 min (age 9), 95 min to 32 min (age 12) and 33 min to 10 min (changes between 9 to 12 years).

**Conclusions:**

Using different non-wear time rules results in significant differences in daily sedentary time and changes in sedentary time. Correcting for wear time appears to be a reasonable approach to limiting these differences and may improve comparability between future studies. Using the 20 min rule, while correcting for wear time, provided the most accurate estimates of sedentary time and changes in sedentary time, compared to the manual reference in 9–12 year-olds.

## Background

There is now substantial interest in the measurement of time spent sedentary (defined as any sitting behavior with an energy expenditure value ≤ 1.5 METs [1]) by children and adolescents, because of the potential for short and long-term health impacts of sedentary time [2], and interventions to modify sitting behavior in children are now underway [3]. Accelerometry is the method of choice for most researchers objectively measuring free-living physical activity and sedentary behavior, including sitting. However, from the onset of choosing accelerometry, several methodological decisions have to be made by the researcher. After the data have been collected, decisions on the definition of non-wear time, minimum wear time to define a valid day, minimum number of valid days and accelerometry cut-points need to be made and all have potential to affect the outcome variables [4, 5].

To date the effect of different cut-points, minimum wear time and minimum number of valid days on physical activity and sedentary behavior outcomes have been examined [6, 7]. However, much less is known about the effect of non-wear time criteria on sedentary time and reported changes of sedentary time. Non-wear time is often defined as counts being equal to, or greater than, a certain amount of consecutive zeros, with only a few studies using self-report diaries to extract non-wear time [8–11]. It has been shown that different non-wear time definitions result in differences in total wear time and the number of non-wear periods [12]. In addition, as sitting often results in zero counts and is most likely to occur in bouts, rather than sporadic episodes, the chosen length of consecutive zeros may also influence the amount of sedentary time reported. Thus far, consecutive zero rules to identify and remove non-wear time range from 10 min of consecutive zeros to 180 min, with or without allowed interruptions and/or using sophisticated algorithms [13, 14]. Consequently, the exact amount of time children and adolescents spend sedentary remains unclear. In addition, using inconsistent rules makes comparison of sedentary time, and changes in sedentary time, between studies very difficult [15].

Accelerometry non-wear time rules might affect sample size and sedentary time, and their associations with health outcomes such as adiposity in different ways [14, 16]. However, the impact of non-wear time rules on sedentary time remains uncertain. Given a probable increase in sedentary time over age [17–19], any impact of non-wear time rules may become even more apparent when looking at changes in sedentary behavior during childhood and adolescence. In addition, it has been shown that patterns of sedentary behavior are different between age groups. Older children and adolescents appear to engage in longer bouts of sedentary behavior compared to younger children [9, 18, 20]. Therefore, longitudinal studies using very stringent rules to identify and remove ‘non-wear time’ (e.g. 10 min of consecutive zeros [8]) may underestimate changes in sedentary time by misclassifying it to a greater degree as non-wear time at follow up. Studies using less stringent rules to define non-wear time (e.g. 60 min of consecutive zeros [9]) on the other hand may overestimate the change in sedentary time. Therefore, the aims of this study were to examine the effect of different approaches to defining non-wear time on sedentary time and changes in sedentary time, and to examine the impact of correcting for wear-time on these differences.

## Methods

Participants were part of the Gateshead Millennium Study birth cohort. Details of this cohort study have been published previously [21]. Participants who had not opted out of the study were contacted to take part in follow up measurements in 2008 and 2010. Measurements for the present study were collected when children were 8–10 years (from here on referred to as age 9) and 11–13 years (from here on referred to as age 12). The study was approved by the Newcastle University Faculty of Medical Sciences Ethics Committee. Informed written consent was obtained from main carer of each child, and children provided their assent to participation.

Children’s height and weight were measured using standardized procedures. Height was measured to the nearest 0.1 cm using a Leicester portable stadiometer (Chasmors, London, United Kingdom). Weight was measured to the nearest 0.1 kg while wearing light clothing using a calibrated electronic scale (Tanita TBF300MA, Chasmors, London, United Kingdom). Body mass index (BMI) was calculated and UK population reference data was used to calculate the BMI z-score for each child [22].

Sedentary behavior was measured using an ActiGraph GT1M (ActiGraph Corporation; Pensacola, Florida) accelerometer. The ActiGraph is a small uni-axial accelerometer which provides valid measures of physical activity and sedentary behavior [23, 24]. Participants were asked to wear the accelerometer on the right hip during all waking hours for 7 days and only remove the monitor during water-based activities, as described previously [11, 25]. Participants and their parents were also asked to record times when the monitor was taken off using a provided log sheet, and briefly note the reasons why the devices were taken off. Data were collected in 15 s epochs and included in the analyses if participants had at least three days with 6 h per day of accelerometry data (more than 80 % of the participants had more than 10 h per day), as a previous study suggested that this provided acceptable reliability for measurement of physical activity and sedentary behavior [26]. In the present study, epochs were defined as sedentary when recorded counts were ≤ 25 counts per 15 s. This Actigraph cut-point has been widely used to define sedentary behavior in adults and children, and has shown good agreement with a posture based monitor when measuring sitting in one study of children [27]. Data between 07.00 h and 23.59 h were used and possible non-wear time during this period was determined using four different rules:

1. Manual screening using the times reported in the self-report diary [11]
2. A sequence of 10 min or more of consecutive zeros [8].
3. A sequence of 20 min or more of consecutive zeros [18].
4. A sequence of 60 min or more of consecutive zeros [9]

Manual data reduction of all accelerometry records took place by comparing non-wear periods (i.e. a string of consecutive zeros) between 07.00 h and 23.59 h with the completed log sheets. Periods of consecutive zeros which were identified as non-wear time according to the log sheets and visually confirmed by a trained researcher were excluded. For the three rules using consecutive strings of zeros, data were excluded if a string of consecutive zeros was equal to or larger than 10, 20 or 60 min. No interruptions were allowed within these time periods.

A custom Microsoft Excel macro was used to calculate sedentary time per day, and the percentage of sedentary time per day for each of the four non-wear time rules. Sedentary time was corrected for wear time differences using the percentage of monitored time which was sedentary time as well as sedentary time in minutes per 12 h day. Minutes per 12 h day were calculated by multiplying the fraction of sedentary time (i.e. sedentary time/wear time) by 720 min (i.e. 12 h). Changes in sedentary time were calculated by subtracting sedentary time at baseline from sedentary time at follow up. As the current study did not include a ‘gold-standard’ of non-wear time, the manual data reduction using log sheets in combination with visual screening of the accelerometer data was used as reference method. This method is less prone to cause differences in sedentary time between age groups and has been used previously as a reference method [28]. Data were tested for normality and differences in sedentary time and the fragmentation of sedentary behavior between the four rules were examined using repeated measures ANOVA with Bonferroni post hoc tests.

## Results

Participant characteristics are described in Table 1. At 9 and 12 years of age, 592 and 508 participants received an accelerometer, resulting in 517 and 440 participants with valid measurements using the most restrictive zero criterion (≥10 min of consecutive zeros). Of the 517 children measured at baseline at age 9, 369 had valid measures at age 12 and were included in the analysis when looking at change in sedentary behavior.

**Table 1:** Participant characteristics at age 9 and 12 y

	9y (*n* = 517)*			12y (*n* = 440)**			9y (*n* = 369)***			12y (*n* = 369)***		
	All participants with valid data			All participants with valid data			Participants with valid data at both 9 y and 12 y			Participants with valid data at 9 y and 12 y		
	Mean	SD	Range	Mean	SD	Range	Mean	SD	Range	Mean	SD	Range
Age (years)	9.3	0.4	8.4 – 10.2	12.5	0.3	11.6 – 13.3	9.3	0.4	8.4 – 10.1	12.5	.3	11.6 – 13.3
Height (cm)	135.6	6.3	116.1 – 154.6	154.6	7.8	129.5 – 176.2	135.2	6.2	116.1 – 154.1	154.7	7.7	129.5 – 174.9
Weight (kg)	33.4	7.5	19.2 – 70.8	49.5	12.0	23.6 – 112	33.0	7.2	19.2 – 67.1	49.6	11.9	23.6 – 112
BMI (kg/m^2^)	18.0	2.9	12.6 – 31.4	20.5	3.8	12.7 – 36.7	17.9	2.8	12.6 – 29.4	20.6	3.8	12.7 – 36.7
BMI-Z score	0.56	1.1	−3.1 – 3.4	0.68	1.2	−4.0 – 3.6	0.51	1.1	−3.1 – 3.4	0.68	1.2	−4.0 – 3.6

-*248 boys and 269 girls; **202 boys and 238 girls; ***168 boys and 201 girls

Average sedentary time and changes in sedentary time are shown in Table 2. For the 517 participants included in the analysis at 9 years of age, mean sedentary time was found to be lowest when using the 10 min zero string rule (364 min per day) and highest using the 6o min zero string rule (426 min per day). Differences between methods were significant (Bonferroni post-hoc *p* < 0.05 for all). For the 440 participants included at age 12 the difference between rules increased, with average sedentary times ranging from 424 min per day to 518 min per day for the 10 min and 60 min zero string rules, respectively. Differences in mean daily sedentary time were significant between all methods (Bonferroni post-hoc *p* < 0.05) except the manual reduction and 20 min zero string rule (Bonferroni post-hoc *p* = 1.00). Mean changes in daily sedentary time between age 9 and age 12 (including 369 participants) ranged from 59 min when using the 10 min rule to 91 min when using the 60 min rule. Differences between methods were significant for all groups (Bonferroni post-hoc *p* < 0.05) except the manual reduction and 60 min zero string rules (Bonferroni post-hoc *p* = 1.0). When sedentary time was adjusted for wear time (i.e. using a 12 h day as described above) differences in mean daily sedentary time between rules decreased from 62 min to 27 min, 95 min to 32 min and 32 min to 9 min for age 9, age 12 and mean changes in sedentary time between age 9 and 12, respectively.

**Table 2:** Wear time and sedentary time in min/day per non-wear time rule

	9 Y (*N* = 517)		12 Y (*N* = 440)		Change (*N* = 369)	
	Mean	SD	Mean	SD	Mean	SD
Wear time						
(min/d)						
Manual	674.3	76.7	709.4	92.6	33.1	93.9
>10 min	674.7^*^	76.8	679.8	95.7	3.3	99.2
>20 min	696.5	77.1	720.4	95.0	22.3	98.4
>60 min	736.9	78.8	774.5	95.4	35.1	101.4
Sedentary time						
(min/d)						
Manual	373.1	63.8	461.6	85.5	87.7	79.0
>10 min	363.6	56.9	423.7	71.7	59.2	70.7
>20 min	385.4	62.9	464.3^*^	81.4	78.2	77.0
>60 min	425.7	67.3	518.2	87.3	91.0^*^	84.2
Sedentary time						
(% of wear time)						
Manual	55.3	7.0	65.1	8.8	9.8	8.8
>10 min	54.0	6.5	62.5	7.6	8.6	7.5
>20 min	55.3^*^	6.8	64.5	7.9	9.2	7.8
>60 min	57.8	6.7	67.0	7.7	9.2^‡^	7.8
Corrected sedentary						
time (min/d)						
Manual	398.3	50.5	468.8	63.4	70.3	63.1
>10 min	388.5	46.7	450.3	54.4	61.7	54.2
>20 min	398.4^*^	48.9	464.6	56.9	66.0	55.9
>60 min	415.8	48.1	482.0	55.7	66.0^‡^	56.0

^*^ not significant compared to manual rule

^‡^ not significant compared to 20 min rule

all others significant (*p* < 0.05)

As shown in Table 2, comparing the zero string rules to the manual rule (treated as a reference in this instance) resulted in significant differences between the manual rule and the 10, 20 and 60 min rules at age 9 (*p* < 0.05). The 10 min zero string rule resulted in the smallest error in mean sedentary time compared to the manual rule (−9.5 min) at age 9. At age 12, the 10 and 60 min rules resulted in significant under and overestimation of mean sedentary time, respectively, compared to the manual rule (*p* < 0.05). The 20 min zero string rule resulted in a small non-significant overestimation (2.7 min) of mean daily sedentary time compared to the manual rule. In addition, when looking at changes in sedentary time the 10 and 20 min rule underestimated mean change in sedentary time compared to the manual rule (*p* < 0.05). However, when using sedentary time corrected for wear time, the 20 and 60 min rules resulted in the closest estimates of change in sedentary time compared to sedentary time determined by the manual data reduction rule at both ages (Table 2).

## Discussion

Analyses showed significant differences in sedentary time and changes in sedentary time between the different non-wear rules. At age 9 years, the 10 min zero string non-wear rule resulted in the closest estimates of sedentary time compared to the manual rule. However, when examining sedentary time at age 12 years and changes in sedentary time, 20 min and 60 min zero-string rule compared best to the manual rule, respectively. This finding is in line by a study done by Chinapaw et al. [12] who suggested 60 min of zero counts was the optimal non-wear time rule in children (mean age 11.7y). When correcting for wear time, the effect of the non-wear rule on sedentary time and changes in sedentary time appeared to decrease. The zero string rule of 20 min of consecutive zeros resulted in the closest estimates to the reference rule at both age 9 and 12 years. This finding is similar to that observed by Esliger et al. [29]. In their study 76 % of 8–13 year olds had zero string bouts greater than 10 min (mean 17.5 min) suggesting a 20 min zero string rule might be appropriate to use in children. Looking at the difference between non-wear rules at both age groups, we noted that differences were larger at an older age. These findings are similar to those reported by Atkin et al. who found differences in sedentary time between non-wear rules ranged from 6 min to 30 min and from 18 min to 54 min at age 9 and age 15, respectively [16].

The increase in differences between non-wear rules at an older age may indicate that the chosen length of zero string counts to define non-wear time becomes more significant at an older age. This is in line with some evidence on changes in patterns of sedentary behavior over time [20]. The changes in patterns of sedentary behavior (i.e. longer bouts of sitting as children or adolescents get older) might be a cause of the larger differences between non-wear rules at an older age. To investigate this further, we analyzed the changes in bouts of sedentary time from age 9 to age 12 (Table 3). These analyses showed an increase in longer bouts of sedentary behavior from age 9 to age 12. Therefore, when using shorter non-wear rules, longer bouts of sedentary behavior may be classified as non-wear time, leading to an underestimation of sedentary time.

**Table 3:** Number of bouts of sedentary behavior per day at age 9 years and 12 years by bout length

	9 Y (*N* = 390)		12 Y (*N* = 390)	
Bouts of sedentary behavior	Mean	SD	Mean	SD
5–9 min	10.1	3.1	13.7	4.3
10–14 min	2.4	1.3	3.7	1.7
15–29 min	1.1	0.9	2.4	1.4
≥30 min	0.2	0.3	0.5	0.6

Difference between age 9 years and 12 years significant for all (p < 0.05)

In line with the findings at both age groups, changes in sedentary time were either underestimated (i.e. 10 min rule) or overestimated (i.e. 20 and 60 min rules) compared to the manual data reduction rule. However, even though the 60 min rule resulted in the largest overestimation of sedentary time at both ages, when examining changes over time, it appeared to provide the closest estimate compared to the manual rule. The difference between the manual and 60 min zero string rules is very similar between the two age groups considered (i.e. an overestimation of 53 min and 57 min per day respectively). This indicates that the amount of non-wear time falsely classified as sitting time is similar at both age groups when using the 60 min rule. This suggests that when using shorter non-wear time rules some of the longer bouts of sedentary time (which appear to happen more often at a later age) are excluded as non-wear time whereas they remain included when using a 60 min rule resulting in closer estimates of change in sitting time when using the 60 min rule.

Differences between non-wear rules decreased significantly in both age groups when correcting for wear time. Results showed that using either of the zero string rules led to a smaller amount of error when compared to the manual rule. The most accurate estimates compared to the manual reference method were found when using the 20 min rule in both age groups. This suggests that correcting for wear time is a reasonable method to adjust for the overestimation of sedentary time which might occur when using a slightly longer zero string rule (i.e. 20 min) at a younger age. However, the underestimation of sedentary time which occurred when using shorter rules appeared to be less affected by controlling for wear time. In addition, when using the corrected sedentary time, the 20 min and 60 min rules provided the most accurate estimates of changes over time. Therefore, when conducting longitudinal studies it may be worth using longer zero-string rules and correcting sedentary time for wear time. This may result in more accurate estimates of sedentary time as well as an improvement in the comparability between studies in future.

The present study had several strengths and limitations. The sample size and longitudinal aspect of the study are strengths. To our knowledge this is the first study examining the effect of non-wear rules on longitudinal data and the first to consider *change* in sedentary time, an important variable to intervention studies and longitudinal studies. In addition, the same data were used in each comparison, which controls for variables other than the non-wear definition. However, it has to be noted that while the 100 cpm cut point provides the closest estimate to true sitting time [27] some standing may be included. This study did not have a ‘gold-standard’ or criterion measure of non-wear time, but manual data reduction using log sheets seems a reasonable reference method, and has been used as such previously [28]. Log sheets and accelerometer data were visually inspected by a trained researcher to reduce any potential measurement error related to the log sheets. In addition, the manual diary as a reference tool was unlikely to cause differences between age groups based on the rules used. This might have been the case if only the zero string rules were used. Also, this study adds to the literature as it indicates the difference between four commonly used non-wear criteria, even without using the manual criteria as a reference method. Several studies have been using algorithms which allow for interruptions of non-wear time. These were not included in the current study as it was decided to include some of the most commonly used and simple non-wear rules [13]. For the inclusion criteria for minimum wear time, it is possible that the results might be slightly different for different days of the week. However, most of the commonly used inclusion criteria for wear-time in the literature specify a certain amount of days without specifying which days should be included. Lastly, the study focused on two specific age groups and therefore generalizability to other age groups may be limited.

## Conclusion

The present study has shown that using different rules to define non-wear time results in significant differences in sedentary time and changes in sedentary time. Whether these differences are large enough to be biologically or clinically significant may depend on the application. However, correcting for wear time appears to be a reasonable approach to limit these methodological differences and improve comparability between studies. Lastly, using a 20 min zero-string rule while correcting for wear time might be the method of choice when measuring sedentary time and changes in sedentary time in 9 and 12 year old children.
